# Unveiling the dynamics: understanding the current scenario and drivers of HIV epidemiology in Pakistan

**DOI:** 10.1186/s12977-025-00668-z

**Published:** 2025-09-19

**Authors:** Akmal Zubair, Muhammad Ali, Safa Wdidi, Adel Qlayel Alkhedaide, Luigi Santacroce

**Affiliations:** 1 Department of Biotechnology, Quaid-i-Azam University Islamabad, Islamabad, Pakistan; 2 Faculty of Medical Sciences Laboratory, Oncology Research Center, University of Shendi, Shendi, Sudan; 3 Department of Clinical Laboratory Sciences, Turabah University College, Taif University, P.O. Box 11099, Taif, 21944 Saudi Arabia; 4 Interdisciplinary Department of Medicine, Sectio of Microbiology and Virology, School of Medicine, University of Bari, Bari, Italy

**Keywords:** HIV, Sex worker, Drug user, Contaminant syringes, Epidemic

## Abstract

Over the past two decades, the global HIV/AIDS pandemic has emerged as one of the most pressing health concerns worldwide. In low-income countries, where resources are scarce, both the prevalence of HIV and the associated mortality rates have been steadily increasing. According to the national AIDS control program, approximately 74,619 individuals in Pakistan are living with HIV/AIDS, with cases distributed across various provinces and autonomous territories. The country’s low literacy rate poses a significant barrier to understanding preventive measures, thereby facilitating the uncontrolled transmission of HIV through sexual intercourse, blood transfusions, and the use of contaminated medical equipment. Vulnerable populations include persons engaged in sex work, transgender individuals, males who have sex with men, and those who inject drugs. The movement and relocation of people, particularly in Karachi, are significant factors in the high prevalence of HIV. In Pakistan, where 20% of drug users are HIV positive the HIV positive population and is still spreading among injection drug users (IDUs). Male sex workers and transgender people who engage in sexual interactions with intravenous drug users are experiencing new outbreaks in some locations. HIV risk behaviors are influenced by limited awareness, social stigma, and inadequate harm reduction programs. The HIV/AIDS epidemic is spreading rapidly throughout Pakistan. This review article aims to identify the various factors that are involved in HIV epidemiology in Pakistan.

## Introduction

**Fig. 1:**
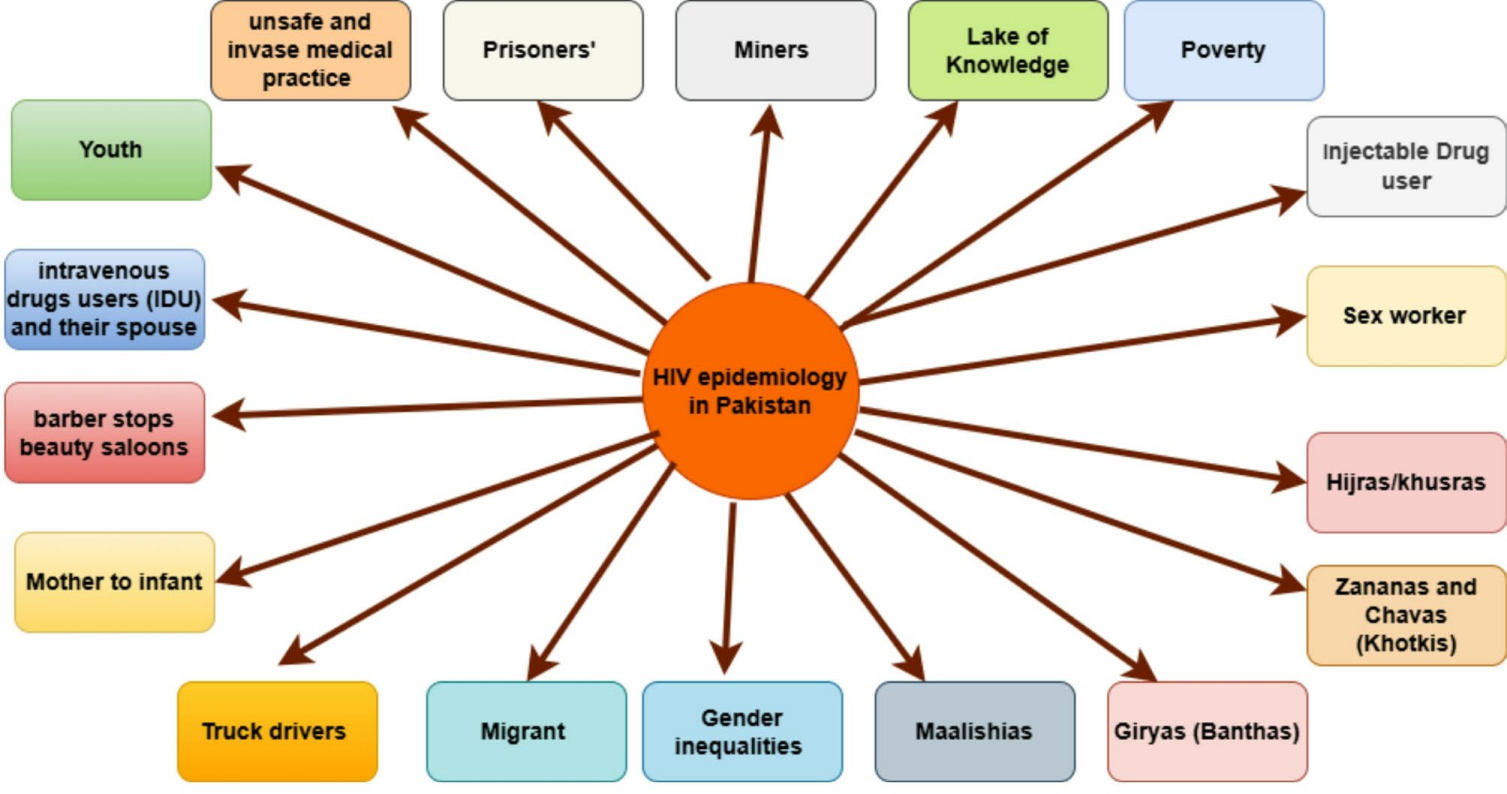
represents the graphical overview of factors behind the emergence of HIV in Pakistan. Some of the terms, such as (Hijras) represent transgenders, ( Zananas and Chavas) represent individuals who are biologically male but identify as female, (Giryas) represent a role of husband upon marrying Hijras and Zenanas, and also work as sex workers. (Maalishias) represent the class of people related massage massage-related therapy, they also act as sex workers.

HIV remains a major global health challenge, with an estimated 39 million people living with the virus worldwide as of 2023, according to UNAIDS. While antiretroviral therapy (ART) has dramatically improved life expectancy and reduced transmission, disparities in access to treatment persist, especially in sub-Saharan Africa, which bears nearly two-thirds of the global burden. In Pakistan, the epidemic has been growing at an alarming rate [1]. The National AIDS Control Program (NACP) reported over 76,167 registered HIV cases by March 2025, but UNAIDS estimates the actual number to be over 210,000, indicating a large proportion of undiagnosed infections. Key drivers include unsafe injection practices, low awareness, poor screening, and stigma surrounding HIV [2]. During the year 1987, Pakistan recorded its first incidence of HIV, which was caused by an improper blood transfusion. [3, 4]. The first outbreak of HIV in Pakistan occurred in 2003-04 among individuals who used injection drugs in rural areas of Larkana, located in the Sindh province. Compared to other countries in the Asia-Pacific region, Pakistan has the second highest rate of AIDS prevalence in last few years [5]. In 2003, the city of Larkana experienced the first significant epidemic of human immunodeficiency virus (HIV) in Pakistan. This outbreak primarily affected people who inject drugs (PWID) [6]. Research conducted during that period among (PWID) revealed that 17 out of 175 individuals, or 9.7%, were confirmed to be HIV-positive [7]. The city faced another HIV outbreak, with infection rates rising beyond initial projections. Patients who were receiving renal dialysis at the dialysis unit of Chandka Medical College are the only ones who were affected by this epidemic [8]. After some time had passed, the National AIDS Control Programme (NACP) conducted an independent investigation. Between September 27 and October 8, 2016, individuals undergoing dialysis were tested for HIV using rapid test kits available under the brand name ImuMed One Step Diagnostic Test [8]. The research conducted by the NACP revealed that the dialysis unit was equipped with eleven dialysis machines, one medical specialist lacking expertise in nephrology, and one technician for each shift. The investigation’s findings indicate a significant deficiency in effective infection control measures, and there is no specialized equipment available for patients infected with HIV, hepatitis B, or hepatitis C [9]. Patients who were interviewed reported that they had received blood from blood banks and laboratories that were unregulated due to their location. According to NACP, access to antiretroviral therapy (ART) remains limited, with only 16% of diagnosed individuals receiving treatment. Pre-exposure prophylaxis (PrEP) is not widely available, and HIV testing services, though offered in government hospitals, private clinics, and NGOs, are underutilized due to stigma and fear of discrimination. The country’s low literacy rate (58%), poverty, and cultural taboos further hinder awareness and prevention efforts. Stigma remains a significant barrier, discouraging people from seeking timely diagnosis and treatment. In this study, we examine an HIV outbreak in Pakistan by highlighting gaps in infection control, drivers of HIV infection in Pakistan, and screening practices to emphasize the urgent need for better prevention strategies and public health interventions in Pakistan. The different factors of HIV are represented in Fig. 1.

According to the results of the fourth round of nationwide surveillance conducted in 2011, the prevalence of HIV was found to be 18.6% among PWID, 15% among hijra (sex workers), 3.1% among male sex workers, and 1.9% among female sex workers. The prevalence of HIV of among age groups are represented in Table 1.

**Table 1:** Represents the prevalence and annual deaths due to HIV. The report is published HIV united nations fact sheet. (WHO, 2020)

HIV in 2020	
Adults (15–49)	0.2 (0.1–0.2)
Adults and children living with HIV	200,000 (190000–210000)
New HIV infections	25,000 (24000–27000)
AIDS-related deaths	8200 (7500–8900)

The infection rates in this small city, located in a rural area and featuring a female brothel along with modest motels that accommodate both male and female sex workers, are astonishingly high [10]. Due to its frequent visits from high-ranking government officials [11]. Table 1 represents the annual deaths and infections of HIV.

## Current situation

NACP has registered 74,619 HIV cases, and among them, 51,821 are on ART in 94 ART centers till December 2024. A prevalence rate of 1.3% was associated with an epidemic discovered in 2018 in the hamlet of Kot Imrana, located within the Tehsil of Kot Momin, Sargodha. The spread of this disease occurred due to untrained individuals reusing needles contaminated with the virus [12]. This rate was subsequently increased to 13% in 2019. In April of that year, a total of 604 children and 135 adults were diagnosed with HIV [13]. The outbreak of HIV in Pakistan is represented in the Table 2.

**Table 2:** Provides an overview of HIV outbreaks in Pakistan from 2000 to 2024

Serial no	Location	Year	References
1	Sargodha District	2007	[14]
2	Kot Imrana, Sargodha District	2018	[15]
3	Jalalpur Jattan, Gujrat District	2008	[16]
4	Chiniot, Punjab	2016	[5]
5	Faisalabad	2019	[17]
6	Larkana, Sind	2003	[18]
7	Larkana, Sind	2016	[19]
8	Ratodero, Sind	2019	[20]
9	Sargodha, Punjab	2019	[6]
10	Dera Ghazi Khan, Punjab	2018	[21]
11	Rawalpindi (Prison Outbreaks)	2021	[5]
12	Nishtar Hospital (Multan)	2024	https://www.dawn.com/news/1874758

**Fig. 2:**
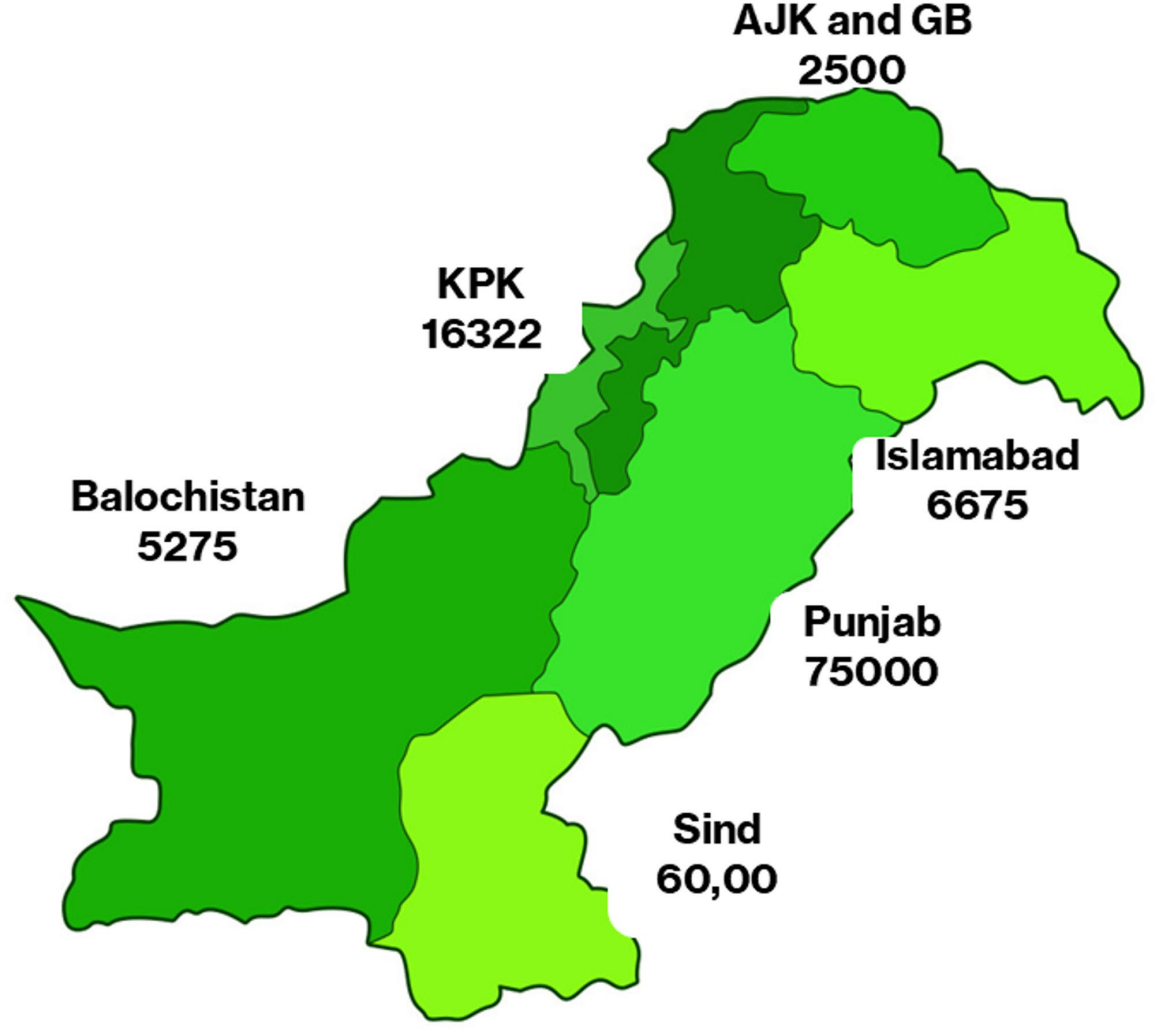
Represent the HIV infection in the provinces of Pakistan

According to the factsheet released by the HIV/AIDS Data Hub for Asia Pacific in 2021, Pakistan has an estimated population of approximately 210,000 individuals living with HIV. This figure includes 41,000 females and 170,000 males. Approximately 4,600 children under the age of 15 years [22]. Figure 2 represents the current situation in Pakistan.

The human immunodeficiency virus (HIV) is a retrovirus that primarily infects CD4 + T cells, with the HIV-1 strain being the most prevalent. HIV is the causative agent that ultimately leads to the development of acquired immunodeficiency syndrome (AIDS) [23]. HIV/AIDS is transmitted from one person to another through the exchange of body fluids, including blood, breast milk, and sperm. Sexual contact, vertical transmission from mother to fetus, breastfeeding, and sharing of syringes are the chief means by which the virus is transmitted [24, 25].

During the past several years, Pakistan has experienced several epidemics, one of which was the Larkana outbreak in 2019 [26, 27].

## Factors behind the HIV epidemics

### Lack of knowledge

The widespread dissemination of false information about this disease has led to the establishment of numerous unfounded beliefs and societal restrictions, severely impeding the progress made in effectively treating HIV. HIV is transmitted from one infected individual to another through the exchange of various bodily fluids, including blood, breast milk, and semen, among other components of the human body [28, 29]. If a mother transmits the virus to her fetus, there is a risk of vertical transmission. However, the people of Pakistan are not well-informed about this issue. According to a poll, only 13% of the population can identify the three primary modes of AIDS transmission, while 50% remain unaware of the existence of AIDS [30]. Most people in the general population believe that the transmission of HIV is solely linked to immoral sexual conduct [31]. Despite this belief, many Pakistanis are under the impression that the spread of HIV due to engaging in unsanctioned sexual behavior is not a significant issue in the Muslim world [32]. It is due to this perception that the range of preventive measures available to address the problem is limited. This limitation allows infections to spread freely through various modes of transmission. It is important to note that the exchange of blood during medical procedures, as well as in barbershops and similar environments, is a significant factor contributing to the spread of HIV [33]. Despite this, they continue to pose a significant threat to society that is often underestimated. The lack of information and awareness in Pakistan is primarily responsible for the high rate of AIDS transmission, which is largely due to the use of needles contaminated with the virus [34]. The high prevalence of illiteracy in Pakistan significantly contributes to the increasing transmission and indiscriminate spread of HIV. Low literacy levels hinder individuals’ ability to understand the preventive measures promoted by healthcare institutions. HIV can be transmitted through sexual contact with an infected partner, extramarital relationships, unsafe blood transfusions, and the use of contaminated needles [35, 36]. A person’s decision to conceal their HIV status due to fear of discrimination from the community may contribute to the spread of HIV. Engaging in high-risk and illegal sexual practices, particularly within the context of prostitution, is another significant factor that facilitates the transmission of HIV [37]. As of 2020, the HIV prevalence rate among the transgender community in Pakistan was estimated to be 5.5%. Due to a lack of clear guidelines and appropriate safety equipment, twenty-three healthcare workforce members are at risk of contracting the virus [38]. The high prevalence of poverty, low literacy rates, and unsafe blood transfusions in Pakistan have all contributed to the country’s increased vulnerability to HIV [34]. The knowledge and practices of medical professionals and students differ significantly, and this distinction is crucial. Patients may hesitate to disclose symptoms during clinic visits due to the burden of admitting to extramarital sexual behavior and the cultural stigma associated with it. This reluctance can lead to humiliation in front of their families and medical professionals [30]. Together with the significant amount of time required for medical treatment, the increasing number of newly diagnosed HIV cases has greatly contributed to this phenomenon [39].

### Injection drug users

It is estimated that approximately 650,000 people in the United States report having a problem with drugs, while the number of individuals who inject drugs (also known as IDUs) may range from 80,000 to 145,000 across the nation [40]. Most of the existing knowledge about injection drug users (IDUs) primarily focuses on males who use drugs in public areas. In contrast, there is significantly less awareness of those who use drugs at home, as well as female users [41]. Before the year 2003, numerous studies indicated that individuals who inject drugs (commonly referred to as IDUs) faced a significant risk of contracting HIV; however, there were no documented cases at that time [42]. Between the ages of 19 and 21, the prevalence of HIV (IDUs) has steadily increased in each of the twelve cities investigated, with the national average hovering around 20% (ranging from 13 to 30%). IDUs often engage in group injections, typically involving five to ten participants, but sometimes larger groups. There is significant variation in the prevalence of syringe sharing among IDUs across different cities, with percentages ranging from 3 to 65% [43]. It is important to note that younger drug users are more likely to engage in syringe sharing. Injections are often administered two to three times per day; however, when heroin is involved, the frequency of injections is typically higher [14]. The percentage of IDUs who have engaged in sexual activity with their regular non-commercial female partners in the past six months is approximately 46%. However, only 10% of these individuals reported using condoms during their sexual activity. In the last six months, a sizeable percentage of persons who inject drugs (IDUs) have participated in paid sexual activity with a female sex worker (FSW), and 13% have done so with male sex workers (MSWs) [44]. However, the frequency of these encounters remains unclear [45]. Some of these occurrences may be already happening, as evidenced by two recently reported outbreaks in Punjab [46]. A continuing investigation into an epidemic revealed that a significant percentage of individuals who developed the illness had received therapeutic injections from local healthcare providers, according to research conducted by the Punjab AIDS Control Program in 2008. Notably, these individuals did not exhibit any of the typical risk factors associated with the transmission of the illness; for instance, they did not engage in sexual activity or use injectable drugs.

### Vulnerable population to HIV

#### Sex workers

A long and illustrious history of prostitution exists in Pakistan [47, 48]. Over the past thirty years, there has been a significant shift in the culture of sex work, moving away from a model primarily focused on brothels toward a more diverse and widespread practice. This evolution includes individuals of all genders—women, men, and transgender individuals (Hijras)—participating in the act of selling sex [49]. It is common for male sex workers (MSWs), transgender individuals known as Hijras, and one-third of all female sex workers (FSWs) to conduct their work in public spaces such as parks or streets. Table 3 illustrates the infection rates among sex workers.

**Table 3:** Represent the sex worker infected with HIV (WHO,2020)

Population	Sex worker	Men who have sex with men (MSM).”	Injectable drug users
Estimated size of population	228,800	832,200	113,400
HIV prevalence %	3.8	3.7	21.0
Know their HIV status	66.7	53.6	21.0
Antiretroviral therapy coverage	5%	0.8%	16.2%

The remaining female sex workers (FSWs) operate their businesses from what they refer to as “kotikhanas,” which can be either their residences or designated places of business. Approximately 3% of individuals are employed in brothels [50]. There is a diverse array of sex workers, including full-time female sex workers and part-time call girls who can be contacted via mobile phone [51]. According to a review of surveillance data, it is estimated that there are approximately 125,000 FSWs across the country. Additionally, there are around 35,000 male sex workers (MSWs) and transgender sex workers. Young people constitute the majority of individuals engaged in the sex trade [52]. The median age of male sex workers (MSWs) is 22 years, while the median age of FSWs and Hijras ranges from 27 to 28 years. MSWs and Hijras often enter the sex trade at a young age, typically between 16 and 17 years, whereas FSWs generally begin their involvement at around 21 years of age [53]. It is estimated that they have an average of seven to ten clients per week. Approximately 50% of individuals rely solely on sex work as their primary source of income. MSWs earn approximately fourteen thousand rupees (50 dollars) per month, while Hijras receive sixteen thousand eight hundred rupees (60 dollars), and female sex workers (FSWs) earn fifty-six thousand rupees (200 dollars) per month [54]. There are only 20% of male-to-female transgender individuals and hijras who report using condoms with their clients, compared to 30% of female sex workers. This trend has remained consistent over the past five years [55].

The findings of a study conducted in 2004, which included 2,400 individuals working in the sex industry, revealed that 58% of Hijras had acquired at least one sexually transmitted infection (STI), while 38% had contracted multiple STIs. Additionally, 62% of the Hijra population in Karachi was found to be suffering from acute syphilis, and 31% were diagnosed with either anal or urethral gonorrhea [56]. 18% of male sex workers (MSWs) were found to have anal gonorrhea, while 36% were diagnosed with syphilis. These findings are consistent with those of the previous study. Additionally, 78% of female sex workers (FSWs) reported experiencing symptoms of sexually transmitted infections (STIs) in the past six months [57]. On the other hand, only 10% of (FSWs) in Karachi and 19% of those in Lahore were currently suffering from any type of sexually transmitted infection (STI). It is important to note that the prevalence of HIV among (MSWs), Hijras, and FSWs was found to be less than 1.5%. According to the surveillance data, there is an increase in the prevalence of STIs among MSWs and Hijras; however, there has been no rise in the prevalence of HIV among FSWs. This variation in prevalence aligns with the findings of the surveillance data [58]. In 2008, the average incidence of male sex workers (MSWs) was 1.1% (ranging from 0 to 3%) across eight cities, while the average occurrence of Hijras was 4.3% (ranging from 0 to 27%) in the same cities [59].

The act of engaging in sexual activity between males is strictly prohibited by religious edicts and social customs, and it is punishable under the Pakistan Penal Code. Nevertheless, a considerable number of men choose to pursue sexual relationships with other men (commonly referred to as MSM) due to the relatively easier access to sexual encounters with other males [60]. This occurs despite societal and religious restrictions that prohibit engaging in sexual activities with women outside of marriage. As a result, many individuals adopt a bisexual lifestyle, which encompasses both sexual experiences with men and socially recognized partnerships with women [61]. The total number of male sex workers (MSWs) and hijras in certain cities is either equal to or exceeds the total number of female sex workers (FSWs). Once HIV becomes established among significant populations of men who have sex with men (MSM) or bisexual individuals, it has the potential to spread rapidly to the general population, particularly among women married to these men [58].

Data from national surveillance indicate that there is a relatively low level of sexual engagement between people IDUs and those working in the sex industry. The percentage of FSWs who have reported having sexual encounters with an intimate partner who is an IDU is only 14%, while just 10% of MSWs or Hijras have reported similar encounters over the past six months [59]. , Ahmad et al. demonstrated that individuals who have been using intravenous drugs (IDUs) for an extended period may exhibit a relatively low level of sexual activity. Nevertheless, those who inject drugs often engage in the exchange of sexual services in return for drugs or monetary compensation [46]. However, the scope of this particular type of commercial sex and its characteristics have not been precisely quantified regarding frequency, network density, and typology of the practice [62]. However, there are variations in patterns that differ from city to city. Cities with a greater number of connections between IDUs and MSWs or hijras tend to exhibit a higher prevalence of HIV [22]. The rate at which HIV spreads from IDUs to sex workers, and potentially to the broader community, will be further influenced by four additional factors. These factors include the cumulative interactions between IDUs and sex workers, the initial prevalence of HIV infection, and the likelihood of drug injection among sex workers themselves [63].

#### Hijras (also known as Khusras)

Hijras is the collective term used in Pakistan for individuals who are transgender, eunuchs, transvestites, hermaphrodites, or intersex. Hijras are typically biological males who may be fully castrated (eunuchs). They often engage in anal sex and are considered to be at high risk for HIV transmission [56].

#### Zenanas and Chavas (Khotkis)

The individuals in question are biologically male but identify as female. To earn an income, they present themselves as women and engage in sexual encounters with various partners. They often enter marital unions with women and have children. When individuals participate in sex work, they adopt the identity and responsibilities typically associated with being a woman. Trans women can redefine sexual roles [64].

#### Giryas (banthas)

These individuals assume the role of a husband upon marrying Hijras and Zenanas, becoming fathers to their children [65].

#### Maalishias

Not only do young and adult men participate in the profession of massage therapy, but they also offer sexual services for a fee. Intravenous drug users (IDUs) represent the highest-risk demographic for acquiring HIV; however, male sex workers are increasingly recognized as the second most vulnerable population [66].

#### Intravenous drug users (IDUs) and their spouses

There are approximately 50% of individuals involved in sex work and intravenous drug use who are also in relationships with other people. The role of spouses and non-commercial partners of sex workers and intravenous drug users in the spread of the pandemic is not fully acknowledged, despite surveillance data indicating that they are vulnerable to HIV [46]. Furthermore, preventive measures have not yet been targeted at this demographic population. There have been studies that a significant proportion of the wives of individuals who inject drugs (IDUs) engage in sex work to supplement their income [40]. However, a recent survey found that only 3% of spouses who inject drugs have ever engaged in sex work [67]. Conversely, it was found that 21% of these women who injected medications (typically diazepam) did so with the assistance of community-based medical practitioners [14]. Using the same injecting equipment that has not been disinfected, these practitioners also administer therapeutic injections to community members as needed [68]. These findings suggest that spouses (IDUs) may facilitate the spread of HIV from IDUs to the general population by sharing syringes with other community members who also receive therapeutic injections from the same healthcare providers. This mode of transmission differs from the more common method of transmission [69].

#### Migrant

Migrants from rural areas often lack the education and work experience of their urban counterparts when they relocate to cities in search of better employment opportunities. They may engage in risky sexual behaviors due to prolonged separation from their loved ones. Without proper protection, sexual contact can transmit HIV to anyone [70]. Rural Punjab and Pakistan’s tribal areas (FATA, Balochistan, and Khyber Pakhtunkhwa) have experienced significant population displacement. All reported cases of HIV in Pakistan involve foreign citizens who have returned from abroad to undergo mandatory testing. A substantial Pakistani diaspora resides in the Gulf countries, with the Middle East hosting just over two million individuals. HIV poses a potential risk of infection to the spouses of these migrants [71].

#### Truck drivers

It is estimated that a significant portion of the population residing in the tribal regions of Pakistan works as truck drivers. They travel to neighboring countries, which are located a considerable distance from their homes [72]. Engaging in sexual intercourse without using protection with coworkers or casual partners during extended periods away from home increases the likelihood of truck drivers developing and transmitting HIV/AIDS [73]. There is a significant number of truck drivers who engage in sexual relationships with young male employed by them. As these young male are often involved in the sex industry, they may participate in sexual encounters with both feminine and masculine individuals. For this specific demographic, sexual engagement typically begins around the age of 17 [74]. They have a marriage rate of 60%, and approximately 25% of them engage in extramarital sexual activity, both for commercial and non-commercial purposes, rarely using condoms [75]. According to the research findings, the prevalence of HIV among truck drivers in Lahore was 1%.

#### Miners

This information is sourced from the Baluchistan AIDS Control Program, which reports that there are currently over 100,000 miners employed across the nation. It has been a significant amount of time since many of them have returned to their homes. A study conducted in a specific region revealed that 42% of workers had engaged in sexual encounters with their colleagues [76].

####  Prisoners

In 2009, the Government of Sindh launched a program designed to provide HIV/AIDS preventive care to prisoners in the cities of Karachi, Hyderabad, and Sukkur. This program offers inmates comprehensive education on the risks of HIV infection and various preventive strategies [77]. To help individuals determine their current HIV status, they can access HIV/AIDS testing and counseling services provided by volunteers in a confidential setting. Individuals aged 10 to 59 and beyond underwent a series of examinations. Out of a total of 4,987 detainees, 49 tested positive for HIV/AIDS, the majority of whom were already diagnosed with the virus [78].

#### The transmission of HIV from mother to infant

It is estimated that approximately 2.3 million children are living with HIV/AIDS worldwide. The vast majority of these young individuals reside in the southern regions of both Africa and Asia [79]. The most common cause of infection in children is the transmission of the virus from mother to child through vertical transmission, which can occur during pregnancy, childbirth, or breastfeeding. Given the increasing number of women and children reported to be contracting the virus from various regions of the country, Pakistan has not escaped the HIV/AIDS pandemic. Although only forty documented cases of parent-to-child transmission have been reported in Pakistan, several indicators suggest that this figure is a significant underestimate from a statistical perspective [80]. Among these challenges are the societal stigma surrounding HIV/AIDS, the limited availability of diagnostic testing facilities, and the difficulties associated with diagnosing children, particularly in a country where more than 30% of children under the age of five are affected by malnutrition. Additionally, another contributing factor is the limited understanding of HIV/AIDS among both the general population and healthcare professionals [81].

#### Barber shops, beauty salons

Barbers are trained professionals who specialize in cutting and trimming beards, as well as various types of hair. They provide shaving services and offer expert haircuts. Unlike hairdressers, who typically focus solely on cutting and styling hair, barbers possess the skills to perform both tasks. Barbering is not the same as a hairdresser since the latter are often only able to cut and style hair.. One of the most time-honored professions in the grooming industry [82]. The earliest historical sources related to barbers indicate that they played significant roles within their respective tribes on numerous occasions. In Nigeria, as well as in other countries across Africa and Asia, the risks of HIV transmission associated with barbering practices have been well-documented [83]. The practice of reusing razors and blades by barbers, combined with a lack of awareness regarding the transmission of HIV, is a significant factor contributing to this issue. A recent study conducted in Karachi, Pakistan, revealed a higher frequency of cuts and nicks during manicure and pedicure procedures [84]. The study was conducted in 250 beauty salons. Due to the vulnerability of these environments, there is an increased risk of developing Hepatitis C Virus (HCV) and possibly Human Immunodeficiency Virus (HIV) [85].

#### Prevalence of HIV in Pakistani youth

Young people in Pakistan, much like their peers in other parts of the world, often find themselves fascinated by sexuality and drugs. This phenomenon is not exclusive to Pakistan. During their teenage years, as they develop their routines and values, they are significantly influenced by those around them [8]. In 2005, a separate study that was carried out by the National AIDS Control Program discovered that the streets of Karachi were the sole place where young people had their first sexual encounter between the ages of 13 and 15 years old, and 30% of those young people had sold sex to both men and women [86]. 80% of individuals do not use condoms, and when they do, the decision to use one is typically made by the more experienced partner rather than the younger partner [40]. Factors such as unemployment, the easy availability of narcotic drugs at low prices, and economic dissatisfaction contribute to this troubling reality [87]. Young individuals are more likely to engage in risky behaviors due to these circumstances, which may increase their risk of contracting HIV [88].

### Unsafe and invasive medical practices

According to research conducted by the World Bank in June 2005, Pakistan has a significant prevalence of medical injections, with an average of approximately 4

-5 injections administered to each individual annually [89]. . Additionally, it is common in medical settings to use needles that have not been properly sanitized. The World Health Organization (WHO) reports that unsafe injection practices are responsible for 62% of hepatitis B infections, 84% of hepatitis C cases, and 3% of new HIV diagnoses [90]. It is important to note that in Pakistan, roadside dentists pose a significant risk of HIV transmission to the general public. According to data from the government of Pakistan, there were 6,761 dentists in the country as of 2006, which is a reasonable number considering the population of approximately 155 million. This information suggests a ratio of one dentist for every 23,000 patients [91]. The lack of access to licensed dentists and the exorbitant costs associated with their services are the primary reasons why individuals seek out unlicensed practitioners operating on the highway. Those who practice dentistry by the side of the road disregard cleanliness regulations and reuse equipment on multiple patients. This pattern of behavior is associated with an increased likelihood of HIV/AIDS transmission [92, 93].

### Poverty

Poverty, a significant factor contributing to the spread of HIV, is one of the most pressing issues currently facing Pakistan in terms of its development [94]. A recent study indicates that the number of individuals living below the poverty line in Pakistan is increasing, with approximately 36 million people affected. Those who are economically disadvantaged not only have limited incomes but also lack access to essential amenities and comforts necessary for leading a life of richness and significance [95].

### Gender inequalities

It is possible that gender inequality, which plays a significant role in the situation, contributes to the ongoing spread of HIV/AIDS in Pakistan. In general, Pakistani women face worse socioeconomic conditions, have less mobility, and possess less decision-making power than Pakistani men [96]. This is particularly true in Pakistan, where various factors contribute to the higher likelihood of HIV transmission among Pakistani women compared to their male counterparts. Women are more susceptible to exposure to HIV due to these circumstances [97]. When comparing literacy rates in Pakistan, 35% of women are literate, while the literacy rate among men is 59%. This disparity is a result of gender inequalities that affect the likelihood of attending school [98].

### Other reasons

When considering the factors of poverty, gender inequality, and low literacy rates present in this context, it is important to recognize that there are other reasons. Because of a variety of other frequent patterns of behavior and risk situations, the general population in Pakistan is particularly vulnerable to HIV/AIDS. This vulnerability is exacerbated by most of the population’s circumstances. For instance, the seldom use of condoms, the presence of filthy settings, and the execution of potentially hazardous medical procedures are all contributing factors [99].

## Conclusion

A significant number of HIV/AIDS cases are currently being reported in Pakistan, particularly among vulnerable populations. The country is facing a challenging situation because of this epidemic. Several factors contribute to the development and spread of the disease, including low literacy rates, engagement in high-risk behaviors, and inadequate healthcare services. The interplay of these factors has led to the widespread transmission of the pandemic. Targeted medicines are very important, particularly for certain populations, such as those who participate in intravenous drug use, persons who are working in the sex industry, and transgender people. Women have a higher risk of contracting HIV than men do because of differences in socioeconomic level and a lower degree of autonomy in decision-making. The prevalence of gender inequality as a contributing factor makes this problem much more pervasive and difficult to solve. The implementation of a comprehensive plan that includes education, healthcare reform, and empowerment efforts is necessary to effectively address these difficulties. These are the fundamental components of the plan. Urgent intervention is required through national policy changes, increased funding for HIV prevention programs, and stronger international partnerships to curb the epidemic and protect vulnerable populations in Pakistan. Pakistan is facing a growing HIV epidemic, and urgent action is needed to control its spread.

## Recommendations

The key to reducing HIV lies in a multi-pronged approach that combines awareness, prevention, treatment, and policy reforms. First, comprehensive education campaigns should be launched to dispel myths and reduce stigma, encouraging people to seek testing and treatment without fear. Free and easily accessible HIV testing centers must be set up, especially in high-risk areas, to ensure early diagnosis and timely treatment. Ensuring that antiretroviral therapy (ART) is widely available and affordable is crucial, as consistent treatment not only improves patients’ health but also reduces the risk of transmission. Additionally, promoting safe sex practices through condom distribution and counseling can play a vital role in prevention. Engaging religious and community leaders to advocate HIV awareness and support can help break cultural barriers and reduce discrimination. Finally, the government must strengthen policies to improve surveillance, research, and healthcare infrastructure, ensuring that resources are directed where they are needed most. A collective effort involving healthcare professionals, policymakers, civil society, and the public is essential to curb the HIV epidemic and protect future generations.

## Data Availability

No datasets were generated or analysed during the current study.
